# Corneal topographic changes after upper eyelid blepharoplasty prior to cataract surgery

**DOI:** 10.1007/s10792-026-04285-3

**Published:** 2026-10-03

**Authors:** Kleopatra Varna-Tigka, Sophia Göhring, Myriam Böhm, Yaroslava Wenner, Thomas Kohnen

**Affiliations:** Department of Ophthalmology, Goethe-University, Theodor-Stern-Kai 7, 60590 Frankfurt am Main, Germany

**Keywords:** Blepharoplasty, Cataract surgery, Refractive surgery, Pentacam, Corneal astigmatism

## Abstract

**Purpose:**

To investigate the effect of upper eyelid blepharoplasty (UEB) on corneal topography in cataract patients using Scheimpflug technology.

**Methods:**

This prospective single-center, longitudinal study included 88 eyes of 45 patients undergoing UEB for dermatochalasis prior to cataract surgery. Scheimpflug imaging (Pentacam®AXL, Wetzlar), was performed preoperatively and at two weeks and three months postoperatively. The primary outcome was change in corneal astigmatism, assessed as the cylindrical component of total corneal refractive power (TCRP). Secondary outcomes included K1, K2, Kmax, Km, astigmatism axis and anterior chamber depth (ACD), front and back Keratometry (FK, BK).

**Results:**

The mean age of 67.0 ± 8.7 years; 67% of patients were female (n = 30) and 33% male (n = 15). At three-months, statistically significant changes were observed in cylindrical TCRP (P = 0.010), K1 (*P* = 0.023), and ACD (*P* = 0.007). Additionally, FK2 showed a statistically significant change at 2 weeks (*P *= 0.021) but not at three-months. F-KMax and BKm were not significantly changed at 2 weeks, but showed statistically significant changes at three-months follow-up (*P *= 0.043 and *P *= 0.010 respectively).

**Conclusion:**

UEB was associated with minor changes in selected corneal topographic parameters and ACD. Although some changes reached statistical significance, their clinical relevance was not established. Repeat corneal measurements after UEB may be considered before cataract or refractive surgery when precise refractive outcomes are intended. These findings do not establish a specific three-month waiting period or demonstrate an effect on intraocular lens (IOL) power calculations or final refractive outcomes.

## Introduction

As the quality of life and standards of living are in a state of constant flux, and life expectancy continues to increase, concerns regarding facial aesthetics are on the rise. Upper eyelid dermatochalasis remains the most common indication for UEB, as excessive skin on the upper eyelid is the primary cause of aesthetic and functional dissonance. In individuals aged 45 years and older, the age – and sex-standardized prevalence of dermatochalasis was higher in men than in women (19.0% vs 14.4%). Dermatochalasis was also strongly associated with increasing age, suggesting that age-related factors play an important role in its development [1–4]. Dermatochalasis of the upper eyelid, in conjunction with blepharoptosis and eyebrow drooping, has the potential to result in a decline in visual acuity [5, 6]. It has been demonstrated that upper dermatochalasis can result in alterations to corneal topography [5–10]. The overhanging skin can result in mechanical obstruction and blockage of light entering the eye, thereby reducing the peripheral visual field [1–3]. The mechanical effect resulting from the weight of redundant skin has the potential to induce topographical alterations in the cornea, which, in turn, may give rise to aberrations [8–10]. Severe dermatochalasis has been validated to be associated with altered corneal biomechanical properties [11]. Consequently, a plethora of studies have shown that following UEB, there is an alteration in parameters such as corneal topography or higher-order aberrations [5–10].

Cataract is a pathological condition that primarily affects elderly individuals and can be treated with cataract surgery, through phacoemulsification technique. UEB and cataract surgery are both among the most frequently performed procedures in ophthalmology [12]. The pressure applied by the upper eyelid skin has the potential to affect the anterior corneal curvature, thereby influencing the intraocular lens power calculation for cataract surgery [13].

The calculation of intraocular lens (IOL) power is of the utmost importance for refractive cataract surgery. In recent years, a significant number of devices have been developed for the precise calculation of intraocular lens power. This development is largely driven by the increasing demand among patients for refractive lens surgery, particularly for the implantation of multifocal intraocular lenses [14]. Therefore, a critical parameter is the accurate determination of the anterior corneal curvature. Previous studies have evaluated alterations in corneal curvature after blepharoplasty; however, in this study the Pentacam®AXL was utilized (Oculus Optikgeräte GmbH, Wetzlar, Germany) to examine and analyze the changes in the aforementioned parameters. The Pentacam®AXL is equipped with an integrated axial length measurement feature, enabling precise intraocular lens (IOL) calculations. As a diagnostic instrument that utilizes Scheimpflug technology to measure the ocular length, measures as well as the anterior and posterior corneal surfaces and the anterior to posterior surface of the crystalline lens.

**Fig. 1:**
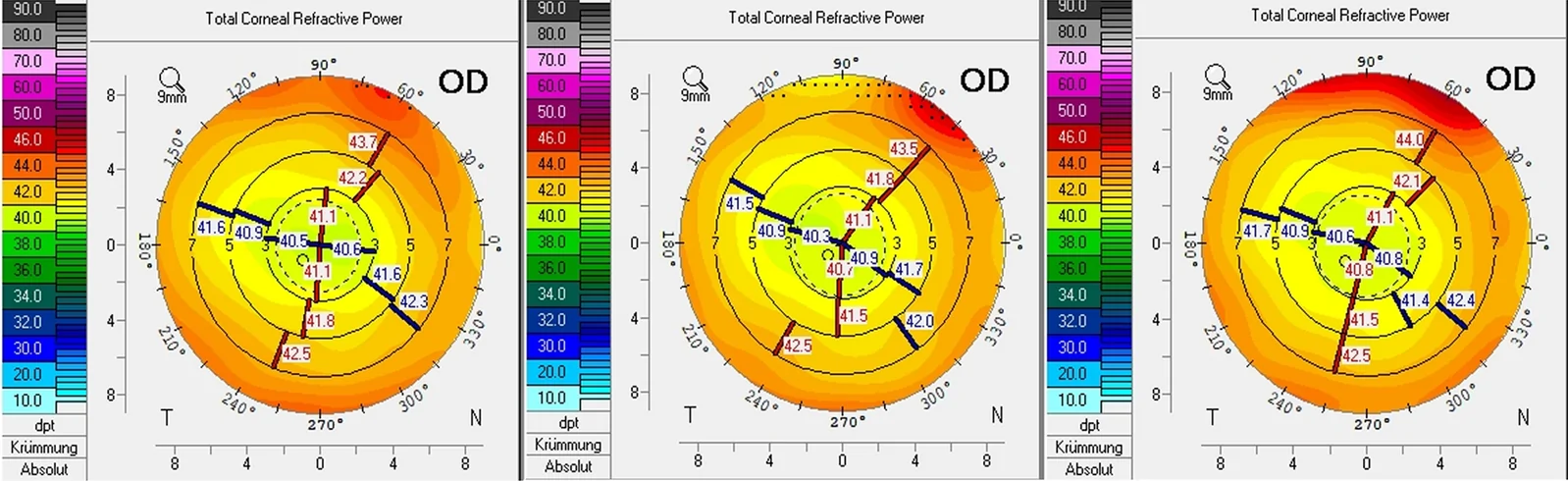
**Representative Scheimpflug tomography (Pentacam®AXL) maps of total corneal refractivepower (diopters) of the right eye (OD) of a patient with cataract and dermatochalasis.** Baseline, 2 weeks and three months after upper eyelid blepharoplasty (UEB). Changes in the corneal power distribution, particularly in the superior cornea, are visible after surgery.

A prospective study was conducted to analyze changes in corneal topography following UEB in patients with cataracts (Fig. 1).

## Methods

Patients were included in a prospective, single-center, longitudinal study from Department of Ophthalmology, Goethe-University Frankfurt, Germany over a period of 12 months, from January to December 2021.

Informed consent was obtained from all participants prior to undergoing UEB, and all protocols were approved by the local University's Clinic Ethics Committee. All methods were carried out in accordance with the tenets of the Declaration of Helsinki.

The inclusion criteria were met by patients with cataracts and dermatochalasis who were scheduled to undergo UEB prior to cataract surgery. Exclusion criteria were corneal surface altering diseases such as keratoconus, pterygium, or contact lens wear; a history of corneal refractive surgery; eyelid pathologies like upper eyelid ptosis, entropion, ectropion, macular pathology, glaucoma, uveitis; and previous ocular or palpebral surgery. A minimum postoperative follow-up period of three months was mandated to ensure the integrity of the study’s findings. All patients included underwent a comprehensive ocular examination, including best-corrected visual acuity (BCVA), slit lamp examination, and fundus examination at the preoperative visit (baseline). Furthermore, patients underwent examination with Pentacam®AXL (Oculus Optikgeräte GmbH, Wetzlar, Germany). Ocular biometry was conducted using the IOL Master 700 (Carl Zeiss Meditec Inc., Dublin, CA, USA).

A total of 45 patients scheduled to undergo UEB were enrolled in the study. At the time of presentation at the department, a detailed history and a routine ophthalmic examination were performed for all included patients. The UEB work-up included measurements of the upper eyelid position, eyelid crease, eyelid fold distance, and eyebrow position. The peripheral visual fields were documented using a 70°-field of view measurement protocol. The palpebral fissure height and eyelid contour were noted as well.

### Surgical technique of UEB

The surgical procedure was carried out by a surgeon with extensive experience in the field (Y.W.), who employed a consistent technique for all patients. The initial step involved marking the surgical area with a blue felt pen. This was followed by the meticulous removal of excess skin using a scalpel and scissors. In cases where the subcutaneous fat pad was prominent, a combination of cautery and scissors was used for its removal. The surgical incision was closed with single button sutures, utilizing 6–0 non-absorbable, sterile surgical sutures (PROLENE™ Sutures). These sutures were removed approximately seven to ten days after the procedure.

### Pentacam®AXL

The Pentacam®AXL is a non-invasive device designed for the analysis of the anterior segment of the eye. The device incorporates a Scheimpflug camera, integrated with a Placido topographer. The device integrates Scheimpflug technology with an interferometry-based biometry technique, capturing slit images from opposing sides of the illuminated slit and calculating the mean elevation data from corresponding images on the opposite side. The Pentacam®AXL provides measurements of several corneal parameters, including corneal curvature (flattest, maximum, steepest, and average) and corneal astigmatism (as measured in the Total corneal refractive power (TCRP) parameter, which is presented on the Cataract evaluation map of the device, in addition to the 4-maps selectable mode). Furthermore, the anterior chamber depth (ACD), the pachymetry at the apex, and the thinnest corneal pachymetry were also analyzed.

### Statistical analysis

Statistical analysis was performed using Microsoft Excel for Mac (Version 16.71, Microsoft, Inc., Redmond, WA, USA) and RStudio (Version 2022.12.0+353, Posit Software, PBC, Boston, MA, USA). The Shapiro–Wilk test was employed to verify the data's normal distribution. For a parametric analysis, the Student´s *t*-test was performed, and for a non-parametric analysis, the Wilcoxon test was used. A p-value of less than 0.05 was considered statistically significant. In the context of this study, both eyes of a single patient were considered as independent units.

The sample-size calculation was based on the primary outcome of change in corneal astigmatism, assessed as the cylindrical component of total corneal refractive power (TCRP) measured with Pentacam®AXL. A study by Simsek et al. [10] evaluating the effect of upper eyelid blepharoplasty on corneal tomographic changes measured by Pentacam®AXL found a mean difference of 0.15 ± 0.5 D in astigmatism. Considering a standard deviation of 0.5, a difference of 0.5 D units of astigmatism was assumed to be clinically significant. Based on these assumptions, 80 eyes were required for a significance level (α) of 0.05 and test power of 0.8 (BiAS for Windows, version 11.01, Epsilon-Verlag).

## Results

A total of 88 eyes from 45 patients diagnosed with dermatochalasis and cataract underwent UEB following a detailed evaluation (30 females and 15 males), with ages ranging from 43 to 83 years (mean age: 67.0 years ± 8.7 years). With the exception of four patients, all subjects underwent a fat pad removal procedure in conjunction with upper lid skin resection. No patient required re-surgery on the upper eyelid during the study period.

### Cataract pre-op mode and total corneal refractive power (TCRP)

Two weeks after UEB, there were no significant changes noted for the flattest (K1), steepest (K2), mean Keratometry (Km) and cylindrical value in total corneal refractive power (TCRP) in the 3 mm zone of the cataract pre-op mode (Table 1a).

**Table 1:** Cataract pre-op mode and total corneal refractive power (TCRP)

(1a)	Baseline		N	2 weeks after UEB		*P* value
	MD	SD	N=88	MD	SD	
K1 (D)	42.19	± 2.03		42.42	±1.92	0.582
K2 (D)	43.11	± 1.65		43.23	±2.13	0.737
Km (D)	42.66	± 1.73		42.83	±1.99	0.333
TCRP (D)	0.91	±1.25		0.81	±0.89	0.731

(1b)	Baseline		N	3 months after UEB		*P* value
	MD	SD	N=75	MD	SD	
K1 (D)	42.47	±1.86		42.66	± 1.69	**0.023**
K2 (D)	43.17	±1.71		43.25	±1.79	0.738
Km (D)	42.83	±1.74		42.96	±1.72	0.157
TCRP (D)	0.71	±0.73		0.59	±0.48	**0.010**

Changes in corneal curvature and total corneal refractive power (TCRP) following upper eyelid blepharoplasty (UEB). No statistically significant changes were observed two weeks after UEB in the flattest keratometry (K1), steepest keratometry (K2), mean keratometry (Km), or cylindrical component of total corneal refractive power (TCRP) in the 3-mm zone (all *P* > 0.05). At three months after UEB, K1 increased significantly from 42.47 ± 1.86 D to 42.66 ± 1.69 D (*P* = 0.023), while the cylindrical component of TCRP decreased significantly from 0.71 ± 0.73 D to 0.59 ± 0.48 D (*P* = 0.010). No significant changes were observed in K2 or Km

*K1* flattest keratometry, *K2* steepest keratometry, *Km* mean keratometry, *TCRP* total corneal refractive power, *SD* standard deviation, *N* number of eyes, *UEB* upper eyelid blepharoplasty

Three months after surgery K1 increased significantly from 42.47 ± 1.86 D to 42.66 ± 1.69 D for K1, and the cylindrical component of TCRP decreased significantly from 0.71 ± 0.73 to 0.59 ± 0.48 (*P* = 0.023 for K1 and *P* = 0.01 for TCRP, Table 1b).

#### Maps selectable mode

A further analysis of the topographic parameters obtained from the 4-map selectable mode was performed. Following parameters were analyzed: Front flattest keratometry (FK1), back flattest keratometry (BK1), front steepest keratometry (FK2), back steepest keratometry (BK2), front maximum keratometry (F-Kmax), front mean keratometry (FKm), back mean keratometry (BKm), front astigmatism (FASTI), back astigmatism (BASTI), pachymetry apex (PApex), thinnest location of the pachymetry (TL), and anterior chamber depth (ACD).

FK2 increased significantly from 43.8 ± 1.67 D to 43.95 ± 1.76 D two weeks after UEB (*P* = 0.021, Table 2a). This change was no longer detectable at the three months follow up (Table 2b).

**Table 2:** 4 Maps selectable mode analysis

(2a)	Baseline		N	2 weeks after UEB		*P* value
	MD	SD	N=85	MD	SD	
FK1 (D)	42.91	±1.97		43.13	± 1.63	0.457
BK1 (D)	−6.0	± 0.65		−6.07	± 0.27	0.262
FK2 (D)	43.8	± 1.67		43.95	± 1.76	**0.021**
BK2 (D)	−6.35	± 0.312		−6.39	± 0.3	0.455
F-KMax (D)	45.37	± 2.33		45.41	± 3.11	0.662
FKm (D)	43.4	± 1.72		43.54	± 1.66	0.474
BKm (D)	−6.15	± 0.553		−6.23	± 0.27	0.054
FASTI (D)	0.915	± 1.28		0.836	± 0.724	0.181
BASTI (D)	0.347	± 0.507		0.316	± 0.214	0.472
PApex (µm)	547.9	± 47.56		549.3	± 34.64	0.858
TL (µm)	535.9	± 51.2		537.0	± 52.28	0.467
ACD (mm)	2.714	± 0.731	N=76	2.787	± 0.7	0.126

(2b)	Baseline		N	3 months after UEB		*P* value
	MD	SD	N=71	MD	SD	
FK1 (D)	43.12	± 1.89		43.30	± 1.62	0.137
BK1 (D)	−6.07	± 0.41		−6.13	± 0.28	0.139
FK2 (D)	43.91	± 1.75		43.92	± 1.65	0.973
BK2 (D)	−6.38	± 0.3		−6.36	± 0.31	0.805
F-KMax (D)	45.15	± 2.35		44.86	± 1.9	**0.043**
FKm (D)	43.51	± 1.73		43.61	± 1.63	0.701
BKm (D)	−6.21	± 0.34		−6.25	± 0.29	**0.010**
FASTI (D)	0.79	± 1.12		0.63	± 0.42	0.192
BASTI (D)	0.3	± 0.29		0.26	± 0.11	0.633
PApex (µm)	551.3	± 28.81		552.7	± 28.82	0.770
TL (µm)	544.8	± 29.48		546.55	± 30.37	0.727
ACD (mm)	2.77	±0.73	N=64	2.86	± 0.68	**0.007**

Changes in anterior and posterior corneal topographic parameters following upper eyelidblepharoplasty (UEB) in the 4-map selectable mode of the Pentacam®AXL. A statistically significant increase in FK2 was observed two weeks after UEB. At three months , F-Kmax and BKm showed statistically significant changes, decreasing from 45.15 ± 2.35 D to 44.86 ± 1.90 D (*P* = 0.043) and from -6.21 ± 0.34 D to -6.25 ± 0.29 D (*P* = 0.010), respectively. ACD also increased signifi cantly from 2.77 ± 0.73mm to 2.86 ± 0.68 mm (*P* = 0.007). No significant changes were observed in the other evaluated parameters

*FK1* Front flattest keratometry, *BK1* Back flattest keratometry, *FK2* Front steepest keratometry, *BK2* Back steepest keratometry, *FKm* Front mean keratometry, *BKm* Back mean keratometry, *FASTI* front astigmatism, *BASTI* back astigmatism, *PApex* pachymetry apex, *TL* Thinnest location, *F-Kmax* Front maximum keratometry, *AXL* Axial length, *N* Number of eyes, *UEB* Upper eyelid blepharoplasty, *ACD* Anterior chamber depth

F-Kmax decreased significantly from 45.15 ± 2.35 D to 44.86 ±1.9 D three months after UEB (*P* = 0.043, Table 2b), while BKm also showed a statistically significant change (*P* =0.010). ACD also showed an increase from 2.77 ± 0.73 to 2.86 ± 0.68 mm three months after UEB (*P* = 0.007) (Table 2b).

## Discussion

As the human lifespan continues to extend and the population continues to age, the primary objective of ophthalmologic practice has evolved to prioritize the enhancement of visual function, thereby enhancing the quality of life for patients. Oculoplastic surgery is not an exception to this overarching concept. Dermatochalasis, characterized by the protrusion of redundant skin and the coverage of the eyes by drooped fat pads, is a hallmark of the aging process. UEB is a surgical intervention that addresses these concerns, aiming to enhance visual acuity and quality of life in the aging population. The influence of the upper eyelid on corneal shape has been well documented [13]. Numerous studies have been conducted to investigate the effect of the eyelid on the shape of the cornea [15] [16].

Brown et al. [15] conducted a prospective evaluation of corneal curvature changes in patients who underwent UEB. A total of 18 corneas from nine patients who underwent blepharoplasty and 24 corneas from 13 patients who underwent ptosis surgery were measured using standard and corneal videokeratography. The study's findings suggest that eyelid repositioning following upper eyelid surgery can lead to visually significant astigmatic changes in the cornea. Despite the limited number of eyes included in the study by Brown et al., our findings align with theirs, indicating a statistically significant astigmatic reduction following UEB.

Zinkernagel et al. [17] also evaluated the effects of upper eyelid procedures on corneal topography and blepharoplasty, differentiating skin-only and additional fat pad removal. A total of 82 eyelids from 43 patients underwent computed corneal topography at baseline and after surgery. The study found that in the skin-only blepharoplasty group (n = 17), there was an astigmatic change of approximately 0.09 D. In contrast, the blepharoplasty group that included fat removal (n = 41) exhibited an astigmatic change of approximately 0.21 D. The researchers emphasized that large fat removal may induce notable corneal curvature change and that the severity of upper eyelid abnormality corresponds with topographical corneal changes. In the present study, analogous findings were validated: an astigmatic change of 0.10 D was observed in 88 eyes in total two weeks after UEB, and an astigmatic change of 0.12 D was observed in 75 eyes in total in the three-month follow-up after UEB. However, the two groups were not separated, as in our study, only four patients underwent UEB without fat removal.

Dogan et al. [8] in their study of 30 patients, reported that UEB appears to induce steepening in the steepest corneal curvature exclusively in patients with some degree of ptosis (i.e., superior margin reflex < 2.5 mm). No topographic alterations were observed in patients with superior margin reflex values greater than 2.5 mm. The study indicated that UEB has an impact on corneal curvature. While the repair of ptosis does influence corneal curvature, our study excluded patients with ptosis. Consequently, our analysis did not consider the influence of eyelid height. The present study focused on the analysis of corneal topography measurements, including K1, K2, Km, TCRP. These parameters were identified from the "Cataract pre-operative mode" provided by the Pentacam® AXL device.

Subsequent analysis of the “4-Maps selectable mode”, as well as other modes available with the Pentacam®AXL, was conducted to ascertain potential alterations in the front and back measurements of the cornea. The analysis revealed an increase in parameters K1, K2, and Km two weeks and three months following UEB. However, only the increase in K1 attained statistical significance (P= 0.023), suggesting that the weight loss of the upper eyelid post-surgery may play a role.

A discrepancy was observed between the preoperative and postoperative TCRP values, as exhibited a continuous decrease until the three-month follow-up (*P*= 0.010).

Comparison of ACD changes revealed that the increase two weeks after UEB persisted at the three-month measurement (*P* =0.007). These findings suggest a possible association between upper eyelid weight loss and UEB. Kocer et al. [18] indicated similar results but according to Ilhan et al. [19] changes in ACD weren’t significant.

Furthermore, an investigation was conducted into the front and back corneal parameters. It was observed that there was a continuous increase in all parameters as measured two weeks and three months after the surgery (*P* > 0.05). A statistically significant increase (*P* = 0.021) was observed in FK2 keratometry two weeks after surgery; however, no such difference was noted at the three-month follow-up, indicating the transient keratometric changes that occur in the early postoperative phase. A comprehensive analysis of anterior and posterior measurements was conducted; however, no statistically significant differences were observed (*P*>0.05).

In summary, the alterations in all parameters confirmed a consistent pattern over the three-month follow-up period. While certain differences among these parameters were found to be statistically significant, no clinically significant differences were observed in this study. The findings of the present study indicate that the reduced gravitational effect on the cornea may induce alterations in corneal curvature and its refractive characteristics, as well as a deepening of the anterior chamber. Given the increasing prevalence of UEBs, it is imperative to acknowledge the potential impact on corneal optical function. These findings may be relevant to patients with UEB considering cataract or refractive surgery, particularly those with pronounced dermatochalasis, high visual expectations, or relevant corneal parameters. A tailored preoperative assessment may help to facilitate appropriate patient counseling. In such cases, clinicians may wish to consider repeat corneal measurement before finalizing IOL calculations. This sequence facilitates the assessment of the appropriate lens power following potential corneal curvature and anterior chamber changes.

Compared with previous studies, the present study includes a relatively larger sample size and incorporates follow-up assessment at 2 weeks and 3 months after surgery. Furthermore, the Pentacam®AXL was used for the measurements, which, to the best of our knowledge, has been rarely reported for this purpose in comparable studies.

### Limitations

This study has several limitations. First, the relatively small, single-center cohort and the absence of a control group may limit the generalizability of the findings. Second, although both eyes of individual patients were included in the analysis, the correlation between fellow eyes should be considered when interpreting the statistical results. Third, most patients underwent concomitant fat-pad removal, whereas a small number underwent skin excision alone; therefore, the individual contribution of each surgical component cannot be determined. Finally, not all patients returned for the three-month follow-up examination, resulting in a smaller number of eyes available for the three-month analysis. In addition, the study did not directly assess whether the observed changes in corneal parameters resulted in clinically relevant changes in IOL power calculation or postoperative refractive outcomes. Furthermore, the 3-month analysis included 75 eyes, which was slightly below the a priori calculated sample size of 80 eyes for the primary outcome. In addition, multiple statistical comparisons were performed without adjustment, increasing the possibility of false-positive findings.

## Conclusion

In this prospective study of patients with cataract and upper eyelid dermatochalasis, UEB was associated with statistically significant changes in selected corneal topographic parameters and anterior chamber depth at two weeks and/or three months after surgery. However, the magnitude of these changes was small, and no clinically significant differences were evidenced in the present study. These findings suggest that corneal measurements may undergo small changes following UEB. Repeat corneal measurements after UEB may therefore be considered before cataract or refractive surgery, particularly when precise refractive outcomes are intended. However, the present study does not establish that a specific three-month waiting period is required, nor does it demonstrate that the observed changes lead to clinically meaningful differences in intraocular lens power calculation or postoperative refractive outcomes. Further prospective studies with larger cohorts, appropriate accounting for correlation between fellow eyes, longer follow-up, and direct assessment of the effect of blepharoplasty -related changes on IOL power calculations and postoperative refractive outcomes are warranted.

## Data Availability

All data generated or analyzed during this study are included in this article. Further enquiries can be directed to the corresponding author.

## Abbreviations

UEB: Upper eyelid blepharoplasty
ACD: Anterior chamber depth
K1: Flattest keratometry
K2: Steepest keratometry
Kmax: Maximal keratometry
Km: Mean keratometry
CA: Corneal astigmatism
A: Axis
TCRP: Total corneal refractive power
PApex: Pachymetry apex
TL: Thinnest location of pachymetry
FK1: Front keratometry
BK1: Back keratometry
FK2: Front steepest keratometry
BK2: Back steepest keratometry
F-Kmax: Front maximum keratometry
FKm: Front mean keratometry
BKm: Back mean keratometry
FASTI: Front astigmatism
BASTI: Back astigmatism
